# Digital health and quality of care in Primary Health Care: an evaluation model

**DOI:** 10.3389/fpubh.2024.1443862

**Published:** 2024-10-29

**Authors:** Ísis de Siqueira Silva, Cícera Renata Diniz Vieira Silva, Claudia Santos Martiniano, Aguinaldo José de Araújo, Renan Cabral de Figueirêdo, Luís Velez Lapão, Renan Cipriano Moioli, Ewerton William Gomes Brito, Severina Alice da Costa Uchôa

**Affiliations:** ^1^Department of Public Health, Postgraduate Program in Collective Health, Federal University of Rio Grande do Norte, Natal, Brazil; ^2^Federal University of Campina Grande, Campina Grande, Brazil; ^3^Department of Nursing, State University of Paraíba, Campina Grande, Brazil; ^4^Department of Public Health, Federal University of Rio Grande do Norte, Natal, Brazil; ^5^IDeas Laboratory, UNIDEMI, Nova School of Science and Technology, Caparica, Portugal; ^6^LASI, Laboratório Associado de Sistemas Inteligentes, WHO Collaborating Centre for Health Workforce Policy and Planing, Nova Instituto de Higiene e Medicina Tropical, Lisbon, Portugal; ^7^Bioinformatics Multidisciplinary Environment, Digital Metropolis Institute, Federal University of Rio Grande do Norte, Natal, Brazil; ^8^Department of Public Health, Postgraduate Program in Collective Health, Postgraduate Program in Family Health, Postgraduate Program in Health Sciences, Federal University of Rio Grande do Norte, Natal, Brazil

**Keywords:** health evaluation, evaluation models, health care quality, access, evaluation, digital health, Primary Health Care

## Abstract

**Introduction:**

The implementation of technologies in Primary Health Care with monitoring and evaluation of the quality of health care is fundamental to direct the access and quality of health care in the context of the Sustainable Development Goals. Our objective was to develop a model for evaluating digital health interventions in primary care, considering its impact on first contact, longitudinality, integrality and coordination in health.

**Methodology:**

This is an exploratory methodological study of a qualitative nature. This study seeks to explore strategic actors’ perceptions of an evaluation model, and was developed in a process between June 2021 and March 2024. The following stages were followed: Identification of previous models available in the literature, model development, model validation, model update. We performed a nominal group consensus technique online with seven experts. Stages taken to define the design of the model: sending the elaborated model, together with an electronic form with 18 subjective questions, such as brainstorming strategy, for recording impressions, judgment of agreement and suggestions; workshop for discussion by videoconference, at the time the objectives and the model diagram were presented, followed by debate with clarification of doubts and suggestions for clarification of the step-by-step design. After incorporating the suggestions, the model agreed upon in the workshop was subjected to another analysis by the same experts, sent in an online Google document, in which possible inconsistencies between the proposed model and the agreed one were verified, as well as the final agreement on the recommended proposal. At the end of this stage, with synchronous and asynchronous remote procedures, consensus was reached.

**Results:**

The proposed evaluation model presents as primary categories, structure, process and result. The structure encompasses four indicators, namely, employees; financial resources; infrastructure resources; and regulatory and strategic resources. The process is organized into three dimensions, namely: technical dimension, organizational dimension and relational dimension. The results will be evaluated in products; intermediate results; and impacts (short, medium and long term). The results will be measured by the seven pillars of quality: efficacy, effectiveness, efficiency, optimization, acceptability, legitimacy, equity.

**Conclusion:**

This study achieved the goal of developing a model to evaluate digital health interventions in Primary Health Care, helping to identify adequate and useful evaluation methods filling the gap of the lack of quality evaluation standards in the brazilian Digital Health Strategy. It presents an important difference in relation to models from different countries, as it considers the impacts on Primary Health Care quality attributes such as first contact care, longitudinality, integrality and coordination of care. The model will be used by managers and health professionals of Primary Health Care in a case study in Brazil to evaluate the quality of health care. It is expected that the proposed model may be used in other evaluation studies and countries through contextual adaptations.

## 1 Introduction

The advancement of digital technologies has motivated the accelerated development of information systems, applications, decision support systems based on artificial intelligence, machine learning, and internet of things, among others (1, 2). In order to improve health care, the use of digital technologies in this sector has expanded, such as wearable devices, computerized decision support systems, and telehealth, which increase the technical performance and satisfaction of health professionals, showing the potential to reduce the direct and indirect costs of health services and improve the access and quality of care provided (3).

The implementation of digital interventions in health has fostered the formulation of the concept of digital health, which is evolving and is characterized as a broad field that encompasses various Information and Communication Technologies (ICTs) applied to healthcare. For instance, artificial intelligence is being used to improve diagnostics and personalized treatments; data analytics tools are assisting in predicting health trends and managing patient data; blockchain ensures secure sharing of medical records; health information systems enable the efficient management of patient information across different levels of care; the internet of things, with integrated sensors allows for real-time monitoring of patients’ vital signs; teleconsultations facilitate remote medical consultations, and telemonitoring helps in managing chronic conditions from a distance (4–6).

Within the scope of Primary Health Care (PHC), defined as the first point of contact of users with the health care network, which offers comprehensive and accessible care (7), technologies can strengthen the communication and continuity of primary care (8, 9). Digital health infrastructure and its integration into PHC services vary between countries, influenced by the economic situation, health priority and technological advances in the region (10). In line with health, the so-called digital transformation process can create opportunities to expand services in the health sector, provided that there is adequate infrastructure and the necessary training, both for professionals and patients (11).

Digital health offers numerous benefits for improving the quality of life and well-being of populations. Firstly, it facilitates access to health information and medical services, particularly in remote regions, through mobile technologies and digital platforms. Additionally, it enables the management and monitoring of chronic conditions via applications and tracking devices, allowing for continuous and personalized patient follow-up. Furthermore, digital health contributes to the efficiency of health systems by reducing operational costs and optimizing resource allocation. Digital interventions also promote patient engagement, encouraging healthy behaviors and increasing adherence to prescribed treatments (4).

The use of digital health presents, in addition to benefits, significant challenges for the quality of care. This phenomenon is exacerbated by the existence of a technological gap that separates countries with advanced digital ecosystems and technological support conducive to the implementation of ICTs in health, from those that have not yet reached sufficient maturity (12). In less technologically developed countries, the lack of adequate digital infrastructure, such as reliable internet networks and technological equipment, and insufficient technical support, such as the absence of professionals trained in, digital technologies hinder both access to and the quality of digital care at the first point of contact most people have with the health system, as well as the integration and sharing of health information between different primary care units and other levels of the health system, such as hospitals and specialized clinics, which are the central roles of PHC. Considering these aspects, in many cases, the challenges focus on the coordination and continuity of Health Promotion Care, which is often limited and fragmented (10, 12).

The implementation of digital technologies in PHC with monitoring and evaluation is essential to direct the quality of health care in the context of the Sustainable Development Goals. The evaluation process is considered as the exercise of measuring, understanding and judging the effects of an intervention, in order to support decision-making, as well as allowing the process of continuous improvement (13, 14). In this context, political and academic agents can act, aiming to guide the improvement of health practices, toward a more favorable condition (15).

Beginning in 2013, Brazil implemented the Health Information System for Primary Care, initiating the digitalization process of PHC (16). The lack of standardization in health data collection and processing procedures spurred the need for a National Health Information and Informatics Policy to guide ICTs actions across the entire Brazilian healthcare system (17). Advancing the implementation of ICTs in healthcare, the digital health strategy was approved in 2017 (18), based on the National eHealth Strategy Toolkit published by World Health Organization (WHO) in 2012 (19). To align the principles and guidelines of the Unified Health System (SUS) with the e-government policy, the Ministry of Health has launched programs to incentivize digitalization, including the Conecte SUS program (20), which aims to integrate citizens health information into an app, and the Program to Support the Informatization and Qualification of Primary Health Care Data (21), which aims to financially support the digitalization of PHC.

With advancements in healthcare technologies, the National Health Data Network (22) was created to enable interoperability and integrate data generated and stored across different information systems. The Health Strategy for Brazil 2028 (23) aims to systematize and consolidate the work carried out over the past decade, as outlined in several key documents, particularly the National Health Information and Informatics Policy (17), published in 2015 and revised in 2020, and the e-Health Strategy for Brazil (18). Corroborating the expansion of digital health, in 2023, the Secretariat of Information and Digital Health was established to support the Ministry of Health in formulating policies and strategies for the implementation of digital health (24).

Brazil has expanded its digital health portfolio by investing in data security (25), information systems, promoting interoperability, and incorporating technologies (22, 26). In this context, the SUS Digital Program and the National Digital Health Maturity Index, based on quantitative indicators of digital maturity, were established (26). The strengthening of digital health programs and regulatory frameworks encourages states and municipalities to implement digital strategies within the scope of SUS. Therefore, alongside the implementation of ICTs, promoting the evaluation of digital health is essential, as it is expected to help identify barriers and facilitators, supporting its growth and development. An adequate evaluation requires a model that thoroughly comprehends the object of evaluation. Developing this model involves planning data collection, verifying available resources, designing activities, and specifying changes and outcomes (27, 28).

The expansion of the application of ICTs in health transcends geographical borders, and different countries have been concerned with evaluating the implementation of digital health services. Some frameworks models are already available, such as the Global Digital Health Index (29), The Global Digital Health Monitor (30), and the Healthcare Information and Management Systems Society (31). These models present relevant criteria for the evaluation of digital health, such as governance, infrastructure, development of computerization systems, training for the use of digital health, privacy and data security, telehealth, remote care and user satisfaction, among others. However, these models do not demonstrate the articulation between resources, work processes through digital means and their influence on results. Even considering the results of technologies, they do not assess their impact on the strengthening of health systems in general and in particular on the essential attributes of PHC, the focus of this study.

Although the frameworks mentioned have the potential to be applied in different scenarios, in the case of Brazil, which has the largest universal public health system in the world (32), there is a need for a scientifically validated evaluation model that, at the same time, contemplates domains of technology evaluation and the impact on PHC as a specific basis of SUS in the context of the Brazilian digital health strategy. That said, this study aims to develop a model for evaluating digital health interventions in primary care, considering their impact on first contact, longitudinality, integrality and coordination in health. In this perspective, it will be useful for managers, health professionals and patients to assess the quality of care mediated by digital health services. The results of this study can be applied in countries with a universal health model, which adopt primary health care, and especially for low- and middle-income countries, which are expanding the use of digital health applications.

## 2 Methodology

**Figure 1:**
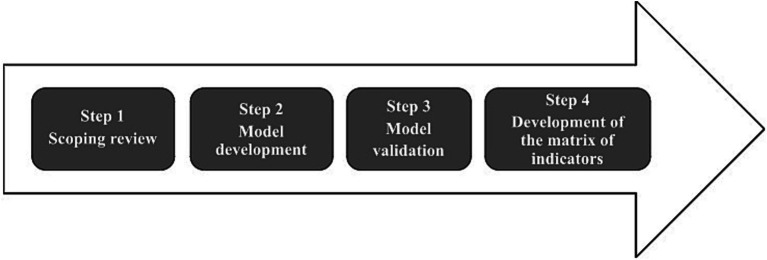
Study steps.

This is an exploratory methodological study that involves the development and validation of tools and methods (33). The present study was qualitative in nature, and its stages are described in Figure 1. This study seeks to explore perceptions of strategic actors (experts) about the model developed between June 2021 and March 2024.

### 2.1 Identification of previous models available in the literature

A scoping review was developed to identify frameworks used to evaluate digital health worldwide during the pandemic. Furthermore, it was the basis for the Brazilian evaluation model. The frameworks are summarized in Table 1.

**Table 1:** Frameworks to evaluate digital health identified in the scoping review (9).

Framework	Purpose	Evaluation
MOMENTUM (61)	To understand the challenges to using telemedicine as a routine service successfully. Identify critical factors in transitioning from a pilot phase to a large-scale use of telemedicine, later integrating it into health care delivery systems.	It considers 18 critical factors for successful use distributed among different domains (strategy, organization, ethics and safety, technological innovation, and market). The critical factors are allocated into four dimensions: (1) context; (2) people; (3) plan; and (4) execution.
Khoja-Durrani-Scott (KDS) Framework for e-Health Evaluation (63)	To provide a comprehensive platform to develop an eHealth evaluation tool.	It describes seven themes to assess in the four stages of the eHealth cycle: development, implementation, integration, and sustained operation. The seven themes are: (1) health services outcomes; (2) technology outcomes; (3) economic outcomes; (4) behavioral and sociotechnical outcomes; (5) ethical outcomes; (6) readiness and change outcomes; (7) policy outcomes.
Commonwealth Scientific and Industrial Research Organization (CSIRO) framework (59)	To outline the delivery, implementation, and evaluation of telehealth services.	Assess and classify the health domain, health services, technology, communication technology, and environmental and socioeconomic settings.
Model for Assessment of Telemedicine (MAST) (58)	To describe the effectiveness and contribution of telemedicine to the quality of care and decision-making processes using a multidisciplinary process. Summarize and systematically and unbiasedly evaluate the information on medical, social, economic, and ethical issues related to telemedicine.	It proposed a three-stage assessment: (1) determining the relevance of an evaluation; (2) results structured in seven domains (targeted health issue; clinical and technical safety; clinical effectiveness; user perspectives including satisfaction, acceptance, usability, access, and self-awareness; economic evaluation addressing costs; changes related to health care use; organizational aspects, including procedural structure, culture, and management aspects; and other sociocultural, ethical, and legal issues); and (3) transferability to understand the potential for expansion.
Clinical, Human and use ICT’s. organizational, Educational, Administrative, Technical, Social evaluation framework CHEATS (62)	To provide a comprehensive framework for evaluating any.	It evaluates clinical, organizational, educational, administrative, and technical aspects and social interactions.
Health Optimum Telemedicine Acceptance Questionnaire (60)	To assess physicians’ perception of the quality of telemedicine services.	It includes eight dimensions for surveying physicians, regardless of their specialty, and focuses on physicians’ perception of the quality of the telemedicine service, its convenience, technical and other difficulties, and potential effects on the health of patients using the service.
Reach, efficacy, adoption, implementation and maintenance (RE-AIM) (64)	To encourage program planners, evaluators, funders and policymakers to pay more attention to essential program elements, including external validity, that may improve the sustainable adoption and implementation of effective and generalizable evidence.	The evaluative dimensions are: Reach the target population; Efficiency; Adoption by the target team; Implementation consistency; Maintenance/sustaining of intervention effects on individuals and environments over time.
Normalization Measure Development (NoMAD) (65)	To evaluate contextual factors that are seen as barriers or facilitators by professionals for the implementation of health interventions.	It focuses on the constructs: Coherence and Cognitive Participation (evaluates the individual and collective involvement of professionals); Collective Action (evaluates the perception of professionals about the implementation in the pre-existing work routine) and Reflective Monitoring (how participants evaluate the new intervention, whether it can be improved and its impact on the daily routine of services).
Global Digital Health Monitor (30)	To monitor digital health progress at the country, regional, and global levels.	The Global Digital Health Monitor is an interactive digital resource that tracks, monitors, and evaluates the use of digital technology for health across countries. The following indicators are considered for evaluation: leadership & governance; strategy & investment; legislation, policy, & compliance; workforce; standards & interoperability; infrastructure; services & applications.

Source: Research data, 2024.

### 2.2 Model development

**Figure 2:**
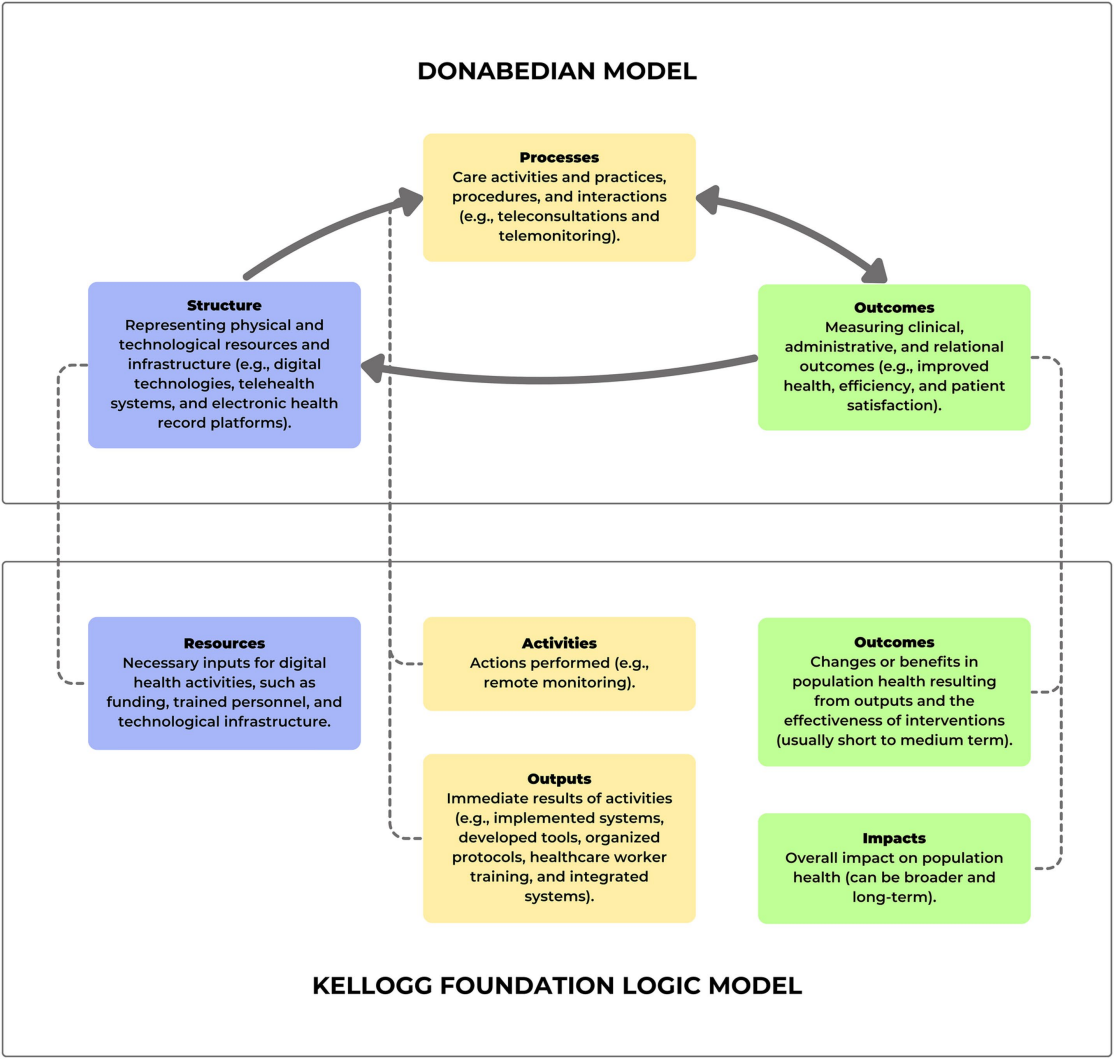
Integration of Donabedian’s model with the Kellogg Foundation’s logic model.

The researchers developed the first version of the model to evaluate Brazilian digital health strategies between September 2021 and January 2022. The integration of Donabedian’s theoretical model (34) and the W. K. Kellogg Foundation’s logic model (35) was adopted to evaluate the quality of care through digital means in a complementary and comprehensive manner. Donabedian’s tripartite model, comprising structure, process, and outcomes, offers simplicity and flexibility, making it widely applicable in various scenarios of health care quality evaluation. Meanwhile, the Kellogg Foundation’s logic model provides a versatile and organized framework, useful for project planning and implementation by incorporating components such as resources, activities, outputs, outcomes, and impacts. The combination of these two models allows for a more detailed analysis, with Donabedian’s model offering a classic and solid foundation for assessing the quality of digital health strategies, while the Kellogg logic model adds greater analytical depth, covering not only immediate outcomes but also long-term impacts. This integration was adapted by the authors and is represented in Figure 2, connecting the technical, organizational, and relational dimensions of digital tools, which are crucial elements to be assessed and improved in PHC.

### 2.3 Model validation

The validation step was a formative moment with broad participation and interaction among members, contributing to the exchange of information and the maturing of opinions. These opinions were based on criticism and systematized ideas, facilitating decision-making with group consensus (36). We performed a nominal group consensus technique (37, 38) online (39) with seven experts, from February to March 2022. The experts were defined based on scientific publications in the areas of health evaluation, digital health, experience in the use of information systems and/or ICTs in primary care, or the development of digital health technologies.

The methodology followed a structured process, comprising four key steps: group preparation, brainstorming, nominal group discussion, and post-group refinement.

*Step 1–Group preparation*: Experts were selected based on their scientific publications in health evaluation, digital health, large experience in information systems, other applications ICTs in primary care, or the development of digital health technologies. Before the nominal group session, participants received the evaluation model (Appendix 1) via email, along with a link to a Google Forms® questionnaire containing guiding questions for the next step, the brainstorming. They could use a pseudonym to ensure confidentiality. This remote format replaced the traditional in-person silent idea generation phase, offering participants more time for reflection.

*Step 2–Brainstormin*: Participants had 10 days to submit their ideas using the electronic form, focusing on identifying gaps in the proposed evaluation model (Appendix 2). All responses were organized in a Microsoft Excel® spreadsheet and shared with the research team for a preliminary analysis, setting the stage for the upcoming nominal group discussion.

*Step 3–Nominal group discussion*: The nominal group discussion was conducted via videoconference using Google Meet®. This method promoted social interaction, democratic discussion, and time efficiency (40). One researcher facilitated the session, ensuring equal time for all participants to present their evaluations on the model components. The themes that emerged during the nominal group are organized in Appendix 3. The experts then clarified and discussed the relative importance of each idea, with a second researcher recording the key points and organizing them into thematic categories.

*Step 4–Post-group refinement*: Following the discussion, the research team incorporated the experts’ feedback into the evaluation model. The revised model was shared with the experts for final verification, ensuring alignment with the group’s suggestions. This iterative process, combining both synchronous and asynchronous inputs, led to a consensus of the model (Appendix 4).

### 2.4 Model update

An additional consultation was necessary due to the end of the global COVID-19 emergency, which directly impacted the practice of care and health and the use of digital health. Between January and March 2024, stakeholders with expertise in primary health care, digital health management and governance were invited to review the design of the digital health model proposed in this research, in order to identify inaccuracies and point out needs for content improvement. The stakeholders were intentionally defined according to their work in health evaluation, digital health, experience in the use of information systems and ICTs in primary care or in the development of digital health technologies, and the invitation was sent by e-mail. Six invitations were sent, among which five accepted to answer the questions, and one accepted, but did not answer the form. The five stakeholders submitted their analyses through an online form that contained the following guiding questions: “What do you consider to be the weaknesses of this model?”; “What do you consider to be the potential of this design?”; “What do you think can be modified?”; “What dimensions of governance need changes?” (Appendix 5).

The responses received were analyzed through thematic analysis and the relevant updates were incorporated into the final structure of the evaluation model.

### 2.5 Ethics and dissemination of the results

The present study was approved by the research ethics committee of the Onofre Lopes University Hospital of the Federal University of Rio Grande do Norte (CAAE 48655521.9.0000.5292). We followed the recommendations of Resolutions 466/12 (41) and 510/16 (42) of the National Health Council and the guidelines proposed by the General Law of Data and Personal Protection number 13.709/2018 (25). All experts signed by e-mail the informed consent form and authorized voice and image recording.

## 3 Results

Before presenting the evaluation model, it is necessary to understand the political context of digital health in Brazil, a summary of the advances in digital health is available in Box 1. The country has stood out as a reference, within Latin America, in the implementation of ICTs in health. During a G20 meeting, a WHO representative highlighted the importance of countries being the protagonists of their own digital transformation and reinforced the Organization’s support, including through Global Digital Health Index, to sustainably improve the health of their populations (43).

BOX 1 **Political-institutional context of digital health in Brazil.**

**Table:** 

Political-institutional context	Year	Description
Telemarketing: TeleSUS (2020), Virtual Family Health Office (2020) and Telehealth Brazil Networks (44)	2011	Institution of the Telessaúde Brasil Redes program, which provides Health Care Network professionals and workers in the SUS with Teleconsulting, Telediagnosis, second formative opinion, and Tele-education services.
Health Information System for Primary Care (SISAB), the operationalization of SISAB occurs through the strategy of the Department of Primary Care called e-SUS Primary Care (e-SUS AB) (16)	2013	It marked an important step in the digitization of processes and medical records, as well as in the standardization of data in primary care.
National Policy on Health Information and Informatics – PNIIS (17)	2015	Its purpose is to promote the innovative, creative, and transformative use of information technology in order to improve health work processes, thereby resulting in a National Health Information System.
e-Health Strategy for Brazil (18)	2017	It established fundamental guidelines for digital transformation in the health sector, promoting the integration of advanced technologies and the interoperability of health information systems.
Support Program for Computerization and Qualification of Primary Health Care Data (21)	2019	It establishes guidelines and procedures for the management of financial resources aimed at data qualification and the digitalization of primary care.
The Health Strategy for Brazil to 2028 (ESD28) seeks to promote and synthesize the necessary revision of the Strategic Vision and the Digital Health Action, Monitoring and Evaluation Plan (PAM&A 2019–2023) for Brazil (23)	2020	It establishes a comprehensive plan for digital transformation in healthcare in Brazil from 2020 to 2028.
National Health Data Network (22)	2020	The document establishes the National Health Data Network and defines interoperability standards to integrate and optimize the exchange of health information between different systems and care units. The ordinance aims to improve the management and efficiency of health data in the country.
Telemedicine actions in the pandemic (45)	2020	Provides for the use of telemedicine during the crisis caused by the coronavirus (SARS-CoV-2).
Guidance for coping with the COVID-19 pandemic (46)	2021	Guidance for coping with the covid-19 pandemic in the Health Care Network, including the use of telehealth.
National System for Digital Transformation and establishes the governance structure for the implementation of the Brazilian Strategy for Digital Transformation (47)	2022	The decree establishes guidelines for digital governance in Brazil, promoting digital transformation and the modernization of public services.
Regulation and institutionalization of the practice of telehealth throughout the national territory (48)	2022	It regulated the practice of telehealth in Brazil.
Creation of the Secretariat of Information and Digital Health – SEIDIGI, responsible for formulating guiding public policies for digital health management (24)	2023	SEIDIGI is responsible for formulating guiding public policies that promote innovation and the adoption of emerging technologies in the health sector.
Update of My digital SUS (49)	2023	Initially called the Conecte SUS Program, Meu SUS digital is providing SUS users with the ability to track their health data, vaccination card, International Certificate of Vaccination or Prophylaxis, authorization for the collection of sanitary products, locate nearby health services, evaluate care, and access other information, thereby facilitating interaction with the health system.
Institution of the SUS Digital Program and the National Digital Health Maturity Index – INMSD (26)	2024	To monitor the progress of SUS digitalization and identify areas that need improvement. The INMSD, which is part of the digital SUS, was created as an assessment tool that measures the degree of digital maturity of health services, with a focus on interoperability, information security and the efficient use of digital technologies. The INMSD, due to its quantitative and standardized nature, although it can offer an important insight into the level of digital maturity in the different regions of Brazil, fails to capture the complexity of local realities, the perceptions of users and health professionals, and the nuances of technological implementations in specific contexts.
Institution of the Inova SUS Digital Laboratory, of the Ministry of Health- an inter-institutional networked, integrative and collaborative environment aimed at promoting, fostering and developing innovative solutions to strengthen the health ecosystem and digital transformation in SUS (50)	2024	To promote a collaborative environment for the development of innovative solutions, strengthening the digital health ecosystem in Brazil.
Source: Research data, 2024

To propose a model to evaluate digital health in the Brazilian PHC, proper understanding and articulation of the model and previous knowledge about it were needed. This knowledge was regarding context, history of digital health, delimitation of the evaluative research, and qualifying indicators of PHC. The involvement of experts in validation has increased the accuracy of the product developed.

During the nominal group discussions, the judges raised potential topics at the intersection of health and technology that required refinement in the initial model (Appendix 3). Among the categories that emerged from the experts’ statements, factors influencing the implementation of digital health were highlighted, such as robust infrastructure and investment in data technologies. Additionally, the issue of healthcare professionals’ training was debated, bringing to light digital health literacy and user engagement. Digital health has the potential to increase the efficiency of PHC and improve universal coverage, but it faces challenges such as data interoperability and the need for effective governance. Considering the political-institutional context and ensuring data ethics and security are also essential aspects for successful implementation. The arguments presented by the judges with expertise in the field were crucial for the model’s development.

**Figure 3:**
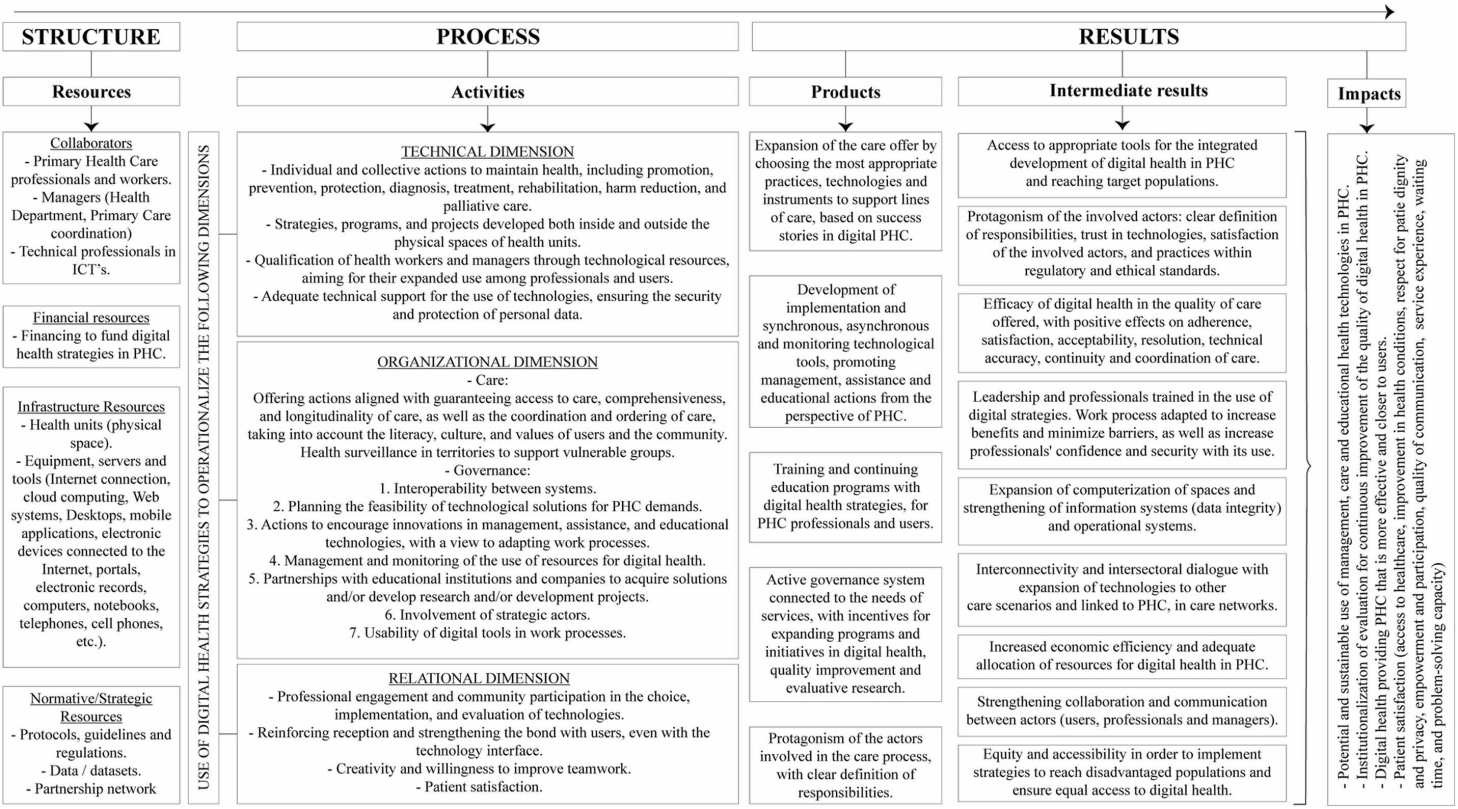
Model to evaluate digital health in Primary Health Care.

The model to evaluate digital health in PHC (Figure 3) comprises different aspects (collaborators, financial, infrastructure, and normative and strategic resources) to allow the execution of activities across the technical (scope of actions and accuracy), organizational (organization of care and management), and relational (interpersonal) dimensions. Given the actions proposed for the implementation strategy, results will be short- (products), medium- (intermediate results), and long-term (impacts).

As presented in Figure 3, the digital health evaluation model in primary health care incorporated elements from Donabedian’s model (34) and the Kellogg Foundation (35).

The structure component incorporates resource elements encompasses four indicators: *employees,* characterized by family health teams, managers and also by Information Technology professionals; *financial resources*, characterized by the financing of digital health in PHC; *infrastructure resources*, characterized by the adequacy of the physical space to perform health care mediated by digital health, computerization (availability of equipment, information systems, internet, applications, electronic devices); and *normative and strategic resources*, characterized by normative regulation of digital health, protocols, guidelines, data storage, and partnership networks.

The process component includes activity elements is organized into three dimensions, namely:

The *technical dimension* that includes the actions of prevention, promotion, protection, diagnosis, treatment, rehabilitation, harm reduction and palliative care, developed in the sphere of PHC; qualification of professionals and users to consciously use digital health, promoting digital health literacy for health professionals, caregivers and patients, health literacy for professionals develop health technologies, technical support for technologies in PHC, and data security.

The *organizational dimension*, which includes the sphere of care (access, comprehensiveness, longitudinality, coordination, ordering and surveillance), governance (governance of systems, technological solutions for PHC demands; encouragement of innovations in managerial, care and educational technologies, with a view to adapting work processes; monitoring the use of resources for digital health; strategic partnerships).

The *relational dimension* is focus on the interaction of health professionals with their patients, and the participation of both in the process of choosing the use of technologies, empowerment of users in health decisions, and strengthening of bonds should be evaluated.

Quality care must maximize the well-being of patients, according to Donabedian (34, 51) the results verify the effects produced in health care, positive results should improve the quality of health care. And the quality applied to health care can be measured by the 7 pillars of quality: *efficacy* (adequate resources), *effectiveness* (ensuring that digital care achieves the same results in everyday practice); *efficiency* (good use of available resources); *optimization* (improvement of the cost–benefit ratio between the amount spent and the result achieved); *acceptability* (adaptation and acceptance of technologies by users and health professionals); *legitimacy* of the ethical and legal compliance of digital care; and *equity* (equal access and quality in digital health services for all people, regardless of their individual characteristics).

The results component is organized into: *Products* (work process mediated by technologies, implementation of ICTs, training for professionals and managers, governance system and involvement of participants); *Intermediate results* (digital inclusion, trust in technologies, and the satisfaction of stakeholders with practices that comply with regulatory and ethical standards, digital health literacy, clear effects of the benefits of the use of ICTs in PHC, improvements in health access, expansion of computerization, interoperability, investment and efficiency of resources for digital PHC, and strengthening of communication between users and health professionals at different levels of care networks); and *Impacts*, according to the Kellogg Foundation (35), can be broader and long-term, addressing the effects of actions on the population’s health. The model presented in this study includes the following impact indicators: potential and sustainable use of managerial, care, and educational digital technologies used in PHC; institutionalization of evaluation for the continuous improvement of digital health quality in PHC; and strengthening and enhancing the effectiveness of PHC. Another important impact of digital health use in PHC is the evaluation of patient satisfaction, which will be assessed with indicators that consider: patient participation in the choice and preference of digital strategies used in health care, satisfaction with treatment and outcomes (respect for patient dignity and privacy; patient empowerment and participation); quality of communication; access to health services (availability of consultations and adequate resources to meet patient needs, geographic proximity of services, accessibility); and patient experience in digitally-mediated care, including health professionals’ empathy, waiting time, ease of communication, and responsiveness to patient concerns.

## 4 Discussion

### 4.1 Principal findings

This study presents an evaluation model of primary health care mediated by digital strategies; its application will be useful for patients, health professionals, managers and technology developers to know the potentialities and limitations when using ICTs in health care, and how these tools interfere with the quality of primary care. The organization of a specific model for PHC will help identify patterns and predict trends, allowing more informed and personalized decision-making, providing insights for the implementation of digital health in health care with a focus on primary care, providing continuous quality improvement.

Encouraging the adoption of digital health has contributed to the diversification of digital strategies in health and computerization of health services (52). The inclusion of technologies is modifying the way of providing health care, generating and storing data (11). Thus, the impact on the quality of health care, from the perspective of the digital age, has aroused the interest of researchers in the area (53–55). An investigation focused on the analysis of digital maturity concluded that greater maturity was associated with the maintenance of the patient’s health record, the screening of patient experience data, the monitoring of the patient’s journey and the mitigation of clinical risk (56).

The evaluation model proposed in this study has a theoretical and methodological foundation aiming at advancing and institutionalizing evaluation processes within the Brazilian PHC. This methodological development of the model was qualified using frameworks similar to worldwide evaluations and validated using a collaborative and participatory strategy. The model was based on a formative perspective, intending to provide information to adjust and overcome issues indicated in the evaluation. Thus, the social contribution of this study relies on this model promoting participatory and comprehensive evaluations centered on the stakeholders and engaging the responders (57).

The various evaluation frameworks mapped out exhibit both similarities and significant differences compared to the evaluation model proposed in this study, which focuses on the quality of services based on two recognized theoretical models (34, 35). Our model offers a holistic and structured assessment of the quality of digital health services, in this sense, it does not focus on just one dimension, but demonstrates the interconnection between the structure and its processes, how these influence the results and impacts on health, considering both the organizational, technical and relational dimensions of care, and especially their impacts on the quality of PHC, emphasizing the implementation and sustainability of technologies over time. In contrast, the mapped models (Table 1), such as MAST (58), CSIRO (59), Health Optimum Telemedicine Acceptance Questionnaire (60), and MOMENTUM (61), concentrate on the evaluation of telemedicine. The CHEATS (62) framework assesses information communication technologies in health, detailing technical, educational, and social aspects. The Khoja-Durrani-Scott (KDS) (63) Framework for e-Health Evaluation provides a structured framework for the overall assessment of digital health services quality, with specific indicators for the stages of the e-health lifecycle, also focusing on the implementation and sustainability of technologies over time. The Digital Health Monitor (30) compares the maturity of digital health across different countries, offering a global overview. The RE-AIM (64) is an evaluation and implementation model not exclusively intended for digital health, as is NoMAD (65), an instrument specialized in the normalization of complex interventions within clinical practice.

The healthcare profile within PHC requires a tailored model for evaluating the quality of care mediated by digital strategies. This is justified because PHC offers philosophical support, as a strategy for organizing and reorganizing health systems (66–70), and has some essential attributes, namely: *first contact*: Offering users accessibility and use of health services, being the preferred gateway; *longitudinality*: it comprises the regular provision of care and its use over time, regardless of the presence of specific problems related to health or the type of problem; *comprehensiveness or integrality*: is one of the pillars in construction of the SUS enshrined in the Federal Constitution of 1988 and has four dimensions: primacy of promotion and prevention actions, attention to the three levels of complexity of medical care, articulation of promotion, protection and prevention actions and a comprehensive approach to the individual and families (68); *coordination of care*: articulation between the various health services and actions, so that they are synchronized and aimed at achieving a common objective, regardless of where they are provided; *orientation to the community*: it refers to the understanding that the health needs of individuals, families and the population are directly related to the social context (70); *centrality in the family*: achieved with the consideration of the context and family dynamics to better evaluate how to respond to the needs of each member and with the knowledge of the members and their health problems (69); *cultural competence*: recognition of the distinct needs of population groups, their ethnic, racial and cultural characteristics, understanding their representations of the health-disease processes (69).

Understanding the nuances of PHC in contemporary practice is critical for effective integration of digital technologies (71). That said, the inclusion of digital health strategies needs to take into account the attributes of PHC (9). Healthcare professionals must be prepared to address the ethical, security, and privacy challenges associated with the use of these technologies, while taking advantage of the benefits they provide to improve the quality and affordability of healthcare. In addition to the analysis of digital health, our study focuses on care itself, evaluating the structure, process and result of actions in primary care. The maturity of digital health will be one of the elements analyzed, within the scope of the quality of health care, but acting as a complement, and not the final target of the evaluation.

One critical aspect that warrants further attention is eHealth literacy, a concept essential for enhancing health outcomes in primary care settings. eHealth literacy refers to the ability of individuals to seek, understand, and use health information from electronic sources effectively. Given the increasing integration of digital health tools in primary care, ensuring that both patients and healthcare professionals possess adequate eHealth literacy is crucial for the success of these interventions. Studies have shown that higher levels of eHealth literacy are associated with better self-management of chronic diseases, improved patient engagement, and more effective use of telemedicine services (72, 73). Moreover, addressing eHealth literacy can help reduce health inequities by ensuring that vulnerable populations—such as older adults and those with lower education levels—can benefit from digital health innovations. As digital tools continue to evolve, it is essential that primary care incorporates strategies to improve eHealth literacy, which can, in turn, lead to more equitable health outcomes (74, 75). Thus, integrating eHealth literacy initiatives into primary care is not only a matter of access but also of optimizing the efficacy and reach of digital health strategies.

The model proposed for evaluating digital health in Brazilian Primary Health Care, which integrates Donabedian’s health quality framework and the Kellogg Foundation’s logic model, has similarities and differences to some studies in the literature. The clearest difference is its practical application to evaluate primary care services that use digital strategies in health care in a country in the Global South. Compared to published studies (76, 77), it can be said that all three studies emphasize barriers that must be overcome for the sustainable implementation of digital health. However, our approach stands out for integrating the logic of the Kellogg Foundation and Donabedian’s theory, providing a more dynamic analysis of interventions. The study by Willis et al. (78) also recognizes the importance of engaging healthcare professionals. However, the proposition we present goes further by incorporating contextual aspects and feedback, promoting local adaptations that are essential for the effectiveness of digital initiatives. We present a clearer framework for assessing the sustainability and long-term impact of digital interventions, which has led to a validated matrix of indicators (79). In this way, the proposed modeling, while in line with the concerns of previous studies, also broadens and updates the discussion on evaluating digital health in PHC.

Assessing differences in national contexts in the digitalization of health is crucial, as cultural and infrastructure differences directly impact the success of actions (9, 11). Quality monitoring contributes to the development and design of new technologies, which should focus on the needs of local communities, breaking the mold of coloniality in digital health (80). Digital health strategies implemented in a sustainable way in PHC and with guaranteed quality improvement can help increase service coverage and assist in health care (81).

A model for evaluating ICT care proposed/starting from the global South strengthens the chorus of the emancipation process of these countries, given the power that big techs have over technologies (80). The results of the application of this model can be used to guide governments in the implementation of public digital health policies aimed at emerging countries, countries with PHC, discuss health data security, technologies customized to the realities of each country, guiding about the weaknesses and strengths of the use of technologies in PHC. Countries in the global South will benefit from this model, and will be able to apply it in health quality assessment within technologies and in the development of health data regulatory infrastructures, health information systems, applications and programs.

### 4.2 Limitations and potentialities of this study

This study had a limitation related to the difficulty in terms of responding to the forms sent out, which prolonged the time intended for data collection, since it adopted virtual and asynchronous data collection, where repeated contacts were made with the judges when the validation forms were not responded to in a certain period. On the other hand, the technique allowed access to geographically distant judges and responses at more opportune times.

Despite the strong theoretical consistency of the instrument, its content was qualitatively validated for use in the context of Primary Health Care in Brazil, a country with continental dimensions and different realities, which limits its degree of reproducibility. However, it can be applied with contextual adaptations, maintaining the addressed analytical dimensions. The scalability of the model is associated with its structure based on two international models (Donabedian and the Kellogg logic model), which enhance its replicability in different contexts. For the application of this framework in other countries, it is important for stakeholders in the interested countries to update it. This update is necessary to capture social determinants of health, cultural factors, and aspects related to the organization of each country’s health system. To achieve this, it is crucial to invite health workers, technology developers, and users of health systems to update the indicators according to the country’s reality.

The potential of this model is related to its application for practical evaluation in health services, based on the organization of an indicator matrix, followed by validated instruments, applicable in various data collection methods such as interviews and questionnaires.

## 5 Conclusion

Evaluating the use of digital health in the quality of primary care is opportune to direct continuous education and training of health professionals; promote transparency in decision-making processes and encourage the participation of all stakeholders; ethical and effective use of digital technologies, data privacy, preparing professionals to deal with the challenges and make the most of the opportunities offered by the digital age. The model presented in this article considers the characteristics of realities of Brazilian health services.

This study achieved the goal of developing a model to evaluate digital health in PHC, helping to identify adequate and useful evaluation methods filling the gap of the lack quality indicators of health care mediated by digital strategies. It presents an important difference in relation to models mapped, as it considers the impacts on PHC quality attributes such as first contact care, longitudinality, integrality and coordination of care. The framework will be used by managers and health professionals of PHC in a case study in Brazil to evaluate the quality of digital health normatively. The results of this evaluation, based on the model, could influence the development of digital technologies, the improvement of ICTs used in PHC, public policies, regulations aimed at digital health and the transformation of the health work process. Also, the Model to evaluate digital health in Primary Health Care may be used in other evaluation studies and countries through contextual adaptations.

## Data Availability

The original contributions presented in the study are included in the article/Supplementary material, further inquiries can be directed to the corresponding author.
