# Supplementary figures and images for: Digital health and quality of care in Primary Health Care: an evaluation model
Source: Front Public Health. 2024 Oct 29;12:1443862. doi: 10.3389/fpubh.2024.1443862 (PMC11580794; doi:10.3389/fpubh.2024.1443862)

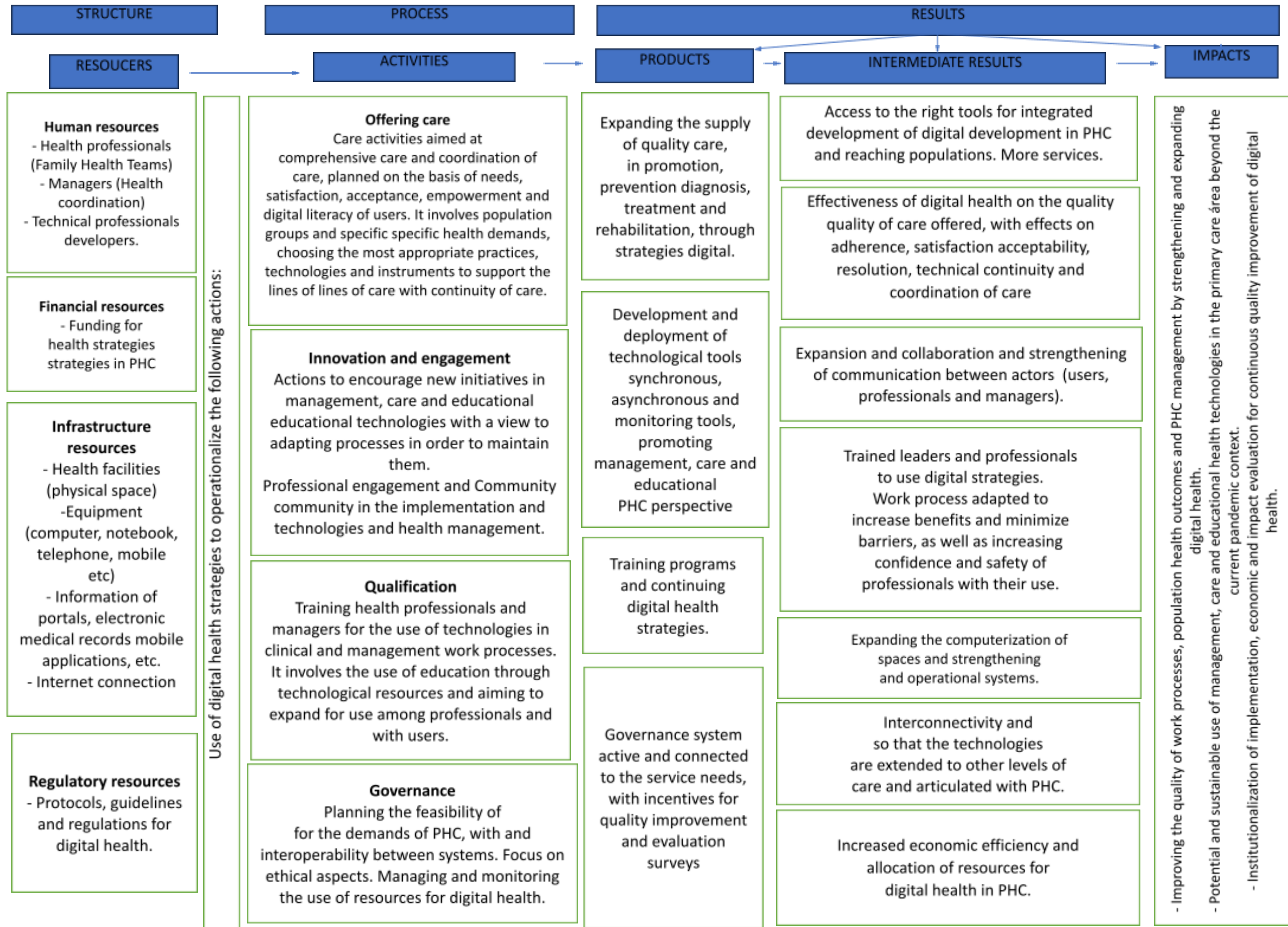

Supplement: Supplementary file 1 [file Data_Sheet_1.PDF]

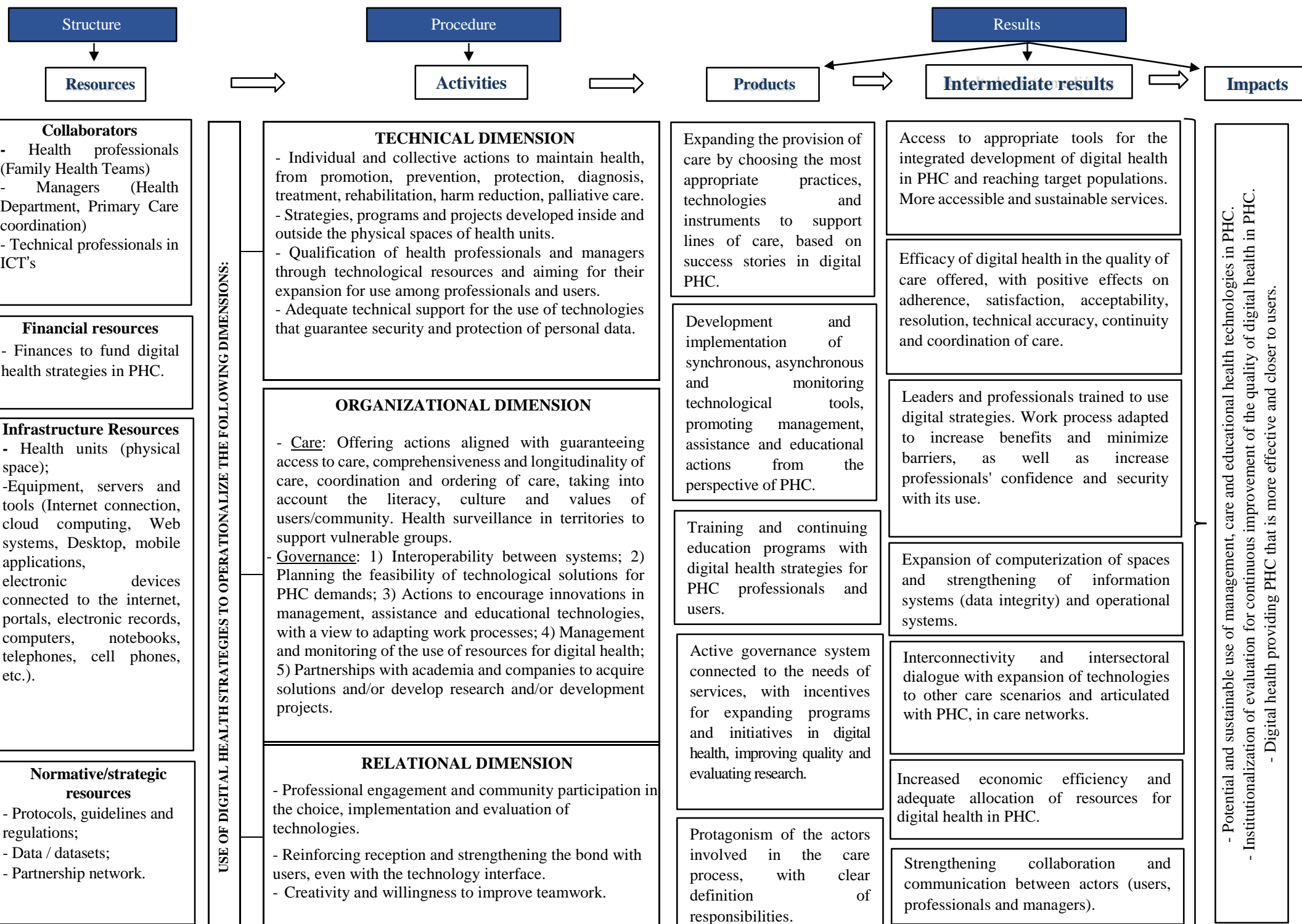

Supplement: Supplementary file 4 [file Data_Sheet_4.PDF]
