# Supplementary material for: Digital health and quality of care in Primary Health Care: an evaluation model
Source: Front Public Health. 2024 Oct 29;12:1443862. doi: 10.3389/fpubh.2024.1443862 (PMC11580794; doi:10.3389/fpubh.2024.1443862)
Supplement: Supplementary file 2 [file Data_Sheet_2.PDF]

Appendix 2. Comments recorded by the judges in the individual analysis stage (google forms) of brainstorming for the nominal group.

**Judges' comments**	
Are the identified frameworks suitable?	J.A.P: “There is a quite broad coverage regarding the analysis of critical success factors and dimensions of application (development, implementation, integration, operation, etc.), in addition to considering aspects both in the health context and in the usability of the systems.”<br><br>J.I.M: “Yes, because as presented in the evaluation, they address important factors for the use of technology in the health field.”<br><br>J.C.S: “Yes, I believe that the frameworks identified in the review are suitable to guide the construction of an evaluation model for the Brazilian context because they had different purposes, providing a varied range of indicators/themes/dimensions evaluated that can be reflected/adapted and, above all, complemented (through the identification of gaps) to fit the local reality.”<br><br>J.O.G: “The chosen frameworks have different purposes with the capacity to structure the digital health evaluation model, engaging with the references used (Donabedian and Kellogg Foundation).”<br><br>J.M.C: “The frameworks that emerged from the literature review provide important and well-founded standardization and guidance for the implementation of the proposed digital health evaluation model in PHC.”
Is there consistency between the frameworks presented and the model drawn up?	J.A.P “I would only add, perhaps in infrastructural resources, data. This will be an extremely important asset for the evolution of computer systems, especially in the clinical and public health management context. Because the lack or poor quality of data is a recurring problem today.”<br><br>J.I.M “I realized that there is coherence with the model prepared, especially in the strategic points of qualification and governance.”<br><br>J.C.S “The model developed contains most of the indicators/dimensions assessed by the frameworks identified in the review.”<br><br>J.O.G “The model presented managed to grasp the evaluative purpose of each framework, which provided pertinent indicators for the proposal.”<br><br>J.M.C “In addition to coherence, it provides originality in the model's propositions.”

How would you rate the Structure component?	J.A.P "The organization's own strategic direction better defines which structures need to be worked on and monitored, including a guideline related to the partnership network and organizational culture. These are items that are within the framework, but I don't see the direct link to where it came from. Therefore, the strategic direction should be added in normative/strategic resources?<br><br>J.I.M "[...] nomenclature on infrastructural resources to be changed to infrastructural resources. I'm not sure if it would fit in this item, but I also missed servers (computers) for running applications. The deployment of tools/solutions can be local or in the cloud (cloud computing)."
Regarding the "Process" component, do you think it is sufficiently representative?	J.I.S "This component lacked a description of activities for monitoring and evaluating digital health actions. Note that the product includes the development and implementation of synchronous, asynchronous and monitoring technological tools."
Do you think the proposed model is relevant for evaluating digital health in Brazilian PHC?	J.A.P "It's already quite complete."<br><br>J.I.M "The model describes key factors for evaluating digital health in PHC in an objective way, using components."<br><br>J.C.S "With the addition of monitoring and evaluation of digital health actions."<br><br>J.O.G "It's important to consider the context in which this model could be used. This is a country with a universal health system, in which PHC is considered the guiding principle of care, but which is suffering major setbacks in its policy, especially in terms of organization and funding. In terms of impact, we should think about how much the use of technology will strengthen PHC from the perspective of the territory, in health promotion and prevention actions."<br><br>J.M.C "Congratulations on your excellent work, which will make a big difference to our healthcare network."
