# Supplementary material for: Digital health and quality of care in Primary Health Care: an evaluation model
Source: Front Public Health. 2024 Oct 29;12:1443862. doi: 10.3389/fpubh.2024.1443862 (PMC11580794; doi:10.3389/fpubh.2024.1443862)
Supplement: Supplementary file 3 [file Data_Sheet_3.DOCX]

Appendix 3. Topics discussed in the synchronous phase of the nominal group

| Discussions addressed in the nominal group | |
| --- | --- |
| **Resources and infrastructure** | - The implementation of digital health depends on a robust infrastructure and adequate resources. - Investments in data science, storage techniques, and machine intelligence are essential for significant future outcomes. - Replace the term “human resources” with “collaborators”. - Normative resources should also include strategic resources. |
| **Engagement and Update of Professionals** | - It is crucial to engage healthcare professionals and update human resources to collaborate in consolidating digital health. - The creation of a dedicated team for technological innovations is necessary to address demands and inquiries. |
| **Donabedian Model** | - Although it is a consolidated model, there is a need to adapt it to the current reality of computing and digital health. - Include and reclassify in the “process” component the technical dimension, organizational dimension, and relational dimension. Adapt these dimensions for the care process mediated by technologies. |
| **Universal Health Coverage and PHC** | - According to the WHO, efficient PHC is essential for universal health coverage; it is important to highlight the relevance of PHC in the context of digital health. - Digital health can make PHC more efficient and expand coverage sustainably. - Digital health should contribute to strengthening PHC and its specific attributes. - PHC needs to be defined in the model, considering its needs and premises in the Brazilian context. |
| **Weakness and Data Interoperability** | - Fragile data and limited interoperability between systems are challenges. - Improving the semantics between data and metadata is essential. |
| **Governance and Monitoring** | - Governance and management must be complemented by effective monitoring. - The use of monitoring through data is crucial. |
| **Structure and Political-Institutional Context** | - PHC needs a strengthened IT structure, acting as the coordinator of the system. - The political-institutional context must be considered in the implementation of the model. - Regional protocols for digital health in Brazil are essential due to regional differences. |
| **Processes and Best Practices** | - Identify successful practices and requirements met by the teams. - Strategic actors involved in healthcare with digital technologies. - Successful cases should be studied and replicated. |
| **Time for development** | - The need for adequate time to develop IT systems in healthcare. - Municipal and state programs with funding should be identified to support development. |
| **Staff Shortage and Consulting** | - There is a shortage of qualified personnel, and hiring consultants may be necessary to select technology and develop resources. |
| **Ethical Aspects and Security** | - The ethics present in traditional healthcare must be transferred to digital health, ensuring the security and privacy of data. - Principles and values of in-person care should be maintained in the virtual environment. |

Source: Research data, 2024.
