# Supplementary material for: Digital health and quality of care in Primary Health Care: an evaluation model
Source: Front Public Health. 2024 Oct 29;12:1443862. doi: 10.3389/fpubh.2024.1443862 (PMC11580794; doi:10.3389/fpubh.2024.1443862)
Supplement: Supplementary file 5 [file Data_Sheet_5.DOCX]

Appendix 5. Update phase of the model in 2024, with stakeholder participation

| **Stakeholder comments** | |
| --- | --- |
| What are the weaknesses of the current model? | Stakeholder 1. "I cannot explicitly see all the digital health evaluation attributes recommended by the WHO."  **Stakeholder 2:** "The model adequately considers the characteristics of the realities of Brazilian healthcare services. A potential weakness may be related to the external validity of the evaluation model if applied in scenarios that diverge from the Brazilian healthcare system, requiring contextual adaptations and validations. Another weakness is that some terms used may be difficult to understand when first encountering the model."  Stakeholder 3. “I cannot perceive any weaknesses”  Stakeholder 4. “The model is complex due to the variety of indicators and dimensions that need to be monitored. This can lead to a workload overload for healthcare professionals and managers. The dependence on financial resources, infrastructure, and specialized IT professionals is significant. In areas with budget constraints or a lack of qualified personnel, implementation may be compromised. The model appears to be inflexible in adapting to different regional contexts. There is a risk of resistance to the use of technology among professionals and patients (users). There is a lack of detail on the mechanisms used to ensure data security and privacy."  Stakeholder 5. "I don't think this is a weakness per se, but there is a need for a local view of PHC and adaptations of the model to different realities. This should be addressed. Another important aspect is the visual presentation of the model. Beyond the article, another form of presentation could be an interactive infographic, for example." |
| What are the potentialities of this design? | Stakeholder 1. “It enables the evaluation of digital health in the context of PHC, differentiating it from other global digital health evaluation models. In addition to evaluating, it allows for the monitoring of digital health actions developed in PHC.”  Stakeholder 2. “The model presents a comprehensive evaluation system. It involves the consolidated components of Donabedian’s evaluation and the dimensions of care quality, in addition to considering the attributes of Brazilian PHC and all actors involved in healthcare through Digital Health (management, professionals, and users).”  Stakeholder 3. “The model is quite comprehensive as it encompasses the classic triad of evaluation: structure, process, and outcome. Additionally, it highlights the relational dimension very effectively.”  Stakeholder 4. "The model has a solid structure based on Donabedian’s theory, which provides a well-organized approach to evaluation. This facilitates the understanding of the key components of digital health in PHC.  By addressing the pillars of quality, the model emphasizes effectiveness, efficiency, acceptability, and other important aspects to ensure that digital healthcare is of high quality. The inclusion of governance and encouragement of innovation can drive the development of creative solutions for digital PHC"  Stakeholder 5. “It provides an expanded view of aspects regarding how digital health can be operationalized to enhance access and quality in PHC, from resources to the intended impacts.” |
| What can be modified? | Stakeholder 1. “I think that in the Organizational Dimension - Governance - the Viability could be included to assess whether digital health functions as intended in the context of PHC, and Usability to check error rates in the use of digital tools in work processes.”  Stakeholder 2. “Clarification of some uncommon and new terms that are not commonly known.”  Stakeholder 3. “In the collaborators, I suggest changing 'health professionals (Family Health Teams)' to 'professionals and workers in Primary Health Care.' This modification will encompass all types of teams and involve other health workers, such as receptionists, who are fundamental in the care process. In the units, there will always be someone to 'open the system' to register the user on the day of the appointment, although any professional can do it. In the technical dimension, in the item regarding the qualification of professionals, 'workers' should also be added."  Stakeholder 4. "Detail how data security and privacy will be ensured, such as through the implementation of rigorous protocols and data protection measures to minimize risks and comply with privacy regulations. Include issues of equity and accessibility in order to implement strategies to reach disadvantaged populations and ensure equality in access to digital health."  Stakeholder 5. “The form of presentation, aside from the publication.” |
| Does the governance dimension need modifications? Which ones? | Stakeholder 1. “It is precisely in the governance section that I think Usability and Viability could be included.”  Stakeholder 2. “Nothing specific.”  Stakeholder 3. “In item 5 of governance, I suggest changing the term 'academy' to 'educational institution.”  Stakeholder 4. “Yes. Promote transparency in decision-making processes and encourage the participation of all stakeholders, including healthcare professionals, managers, IT professionals, and patients (users).”  Stakeholder 5. “Governance: Item 4 - I suggest specifying which resources. Would it only be financial? Item 5: to avoid the repetition of the word 'development,' I suggest using 'increment' in research projects and the development of products/technologies.” |

Source: Research data, 2024.
